# X-linked sideroblastic anaemia in a female fetus: a case report and a literature review

**DOI:** 10.1186/s12920-021-01146-z

**Published:** 2021-12-20

**Authors:** Diane Nzelu, Panicos Shangaris, Lisa Story, Frances Smith, Chinthika Piyasena, Jayanthi Alamelu, Amira Elmakky, Maria Pelidis, Rachel Mayhew, Srividhya Sankaran

**Affiliations:** 1 Guy’s & St. Thomas’ Hospital NHS Foundation Trust, Westminster Bridge Road, London, SE1 7EH UK; 2 Department of Women and Children’s Health, School of Life Course & Population Sciences, Faculty of Life Sciences and Medicine, King’s College London, 10th Floor North Wing St Thomas’ Hospital, London, SE1 7EH UK; 3 Viapath at King’s College Hospital, Bessemer Wing, Denmark Hill, London, SE5 9RS UK

**Keywords:** ALAS2 mutation, Sideroblastic anaemia, X-linked

## Abstract

**Background:**

X-linked sideroblastic anaemia (XLSA) is commonly due to mutations in the *ALAS2* gene and predominantly affects hemizygous males. Heterozygous female carriers of the *ALAS2* gene mutation are often asymptomatic or only mildly anaemic. XLSA is usually characterized by microcytic erythrocytes (reduced mean corpuscular volume (MCV)) and hypochromia, along with increased red cell distribution width. However, in females with XLSA the characteristic laboratory findings can be dimorphic and present with macrocytic (elevated MCV) in addition to microcytic red cells.

**Case presentation:**

We report a case of fetal anaemia, presenting in the early third trimester of pregnancy, in a female fetus. Ultrasound findings at 29 weeks were of cardiomegaly, prominent umbilical veins, a small rim of ascites, and mean cerebral artery peak systolic velocity (PSV) value above 1.5 Multiples of the Median (MoM). She underwent non-invasive prenatal testing that determined the rhesus genotype of the fetus to be rhesus B negative. No red blood cell antibodies were reported. Other investigations to determine the underlying cause of fetal anaemia included microarray comparative genomic hybridization, serology to exclude congenital infection and a peripheral blood film and fetal bilirubin to detect haemolysis. The maternal grandmother had a history of sideroblastic anaemia diagnosed at the age of 17 years. The mother had mild macrocytic anaemia with haemoglobin of 10.4 g/dl and MCV of 104 fl. The fetal anaemia was successfully treated with two in utero transfusions (IUTs), and delivery occurred via caesarean section at 37 weeks of gestation. The red cell gene sequencing in both the mother and fetus were heterozygous for an *ALAS2* mutation causing in utero manifestations of XLSA. The haemoglobin on discharge to the local hospital at five days of age was 19.1 g/dl. Subsequently, the infant became anaemic, requiring regular 3–4 monthly blood transfusions and demonstrating overall normal development. Her anaemia was unresponsive to pyridoxine.

**Conclusions:**

This is one of four cases reporting multiple female members presenting with discordant clinical features of XLSA from being entirely asymptomatic to hydropic in utero. Our report is novel in that there are no previous cases in the literature of anaemia in a female fetus heterozygous for *ALAS2* mutation.

## Background

Congenital sideroblastic anaemias (CSA) are a heterogeneous group of rare disorders. They can be distinguished into syndromic and non-syndromic forms [1]. The syndromic forms include non-haematological manifestations in multiple organ systems and are much less frequent than the non-syndromic forms. The non-syndromic form of CSA can be X-linked or autosomal recessive. X-linked sideroblastic anaemia (XLSA) is the most common, accounting for approximately 40% of cases and is caused by mutations in the *ALAS2* gene [2]. *ALAS2* encodes the enzyme called 5'-aminolevulinate synthase two or erythroid ALA-synthase. This catalyzes the first and rate-limiting steps in the heme biosynthesis pathway in erythroid cells. XLSA is primarily observed in heterozygous males, whereas heterozygous female carriers are often asymptomatic or only mildly anaemic. Autosomal recessive CSA is the second most frequent non-syndromic form of CSA and is caused by mutations in the mitochondrial carrier family gene *SLC25A38* [1, 3].

Non-syndromic CSAs are characterized by erythrocyte microcytosis (reduced mean corpuscular volume (MCV)) and hypochromia, along with increased red cell distribution width.

However, in females with XLSA the characteristic laboratory findings can be dimorphic and present with macrocytic (elevated MCV) in addition to microcytic red cells. Females with XLSA are a significant exception to these characteristic laboratory findings and conversely present with either a normal MCV or erythrocyte macrocytosis (elevated MCV). In severely affected females, macrocytic erythroid cells with the non-functional *ALAS2* enzyme fail to develop into viable erythrocytes and are released at an accelerated rate into the circulation in response to anaemia [1]. Of at least twenty [4–16] unrelated XLSA females, for which sufficient clinical data has been published, seven had microcytic, hypochromic RBCs and were pyridoxine responsive [4, 10, 11, 14–16]. An additional two patients [16] had low-normal MCV and were pyridoxine responsive and thus likely also had a small population of microcytic cells. A variety of gene expression is inherent in the diversity of ALAS2 mutations that number over 80 -particularly in women; who exhibit random X-inactivation as well as skewing of X-inactivation with age and thus, not infrequently, are or become more than mildly affected [17].

We report the first case of fetal anaemia in a female fetus where genetic analysis confirmed heterozygosity for an *ALAS2* gene mutation causing X-linked CSA.

## Case presentation

**Fig. 1:**
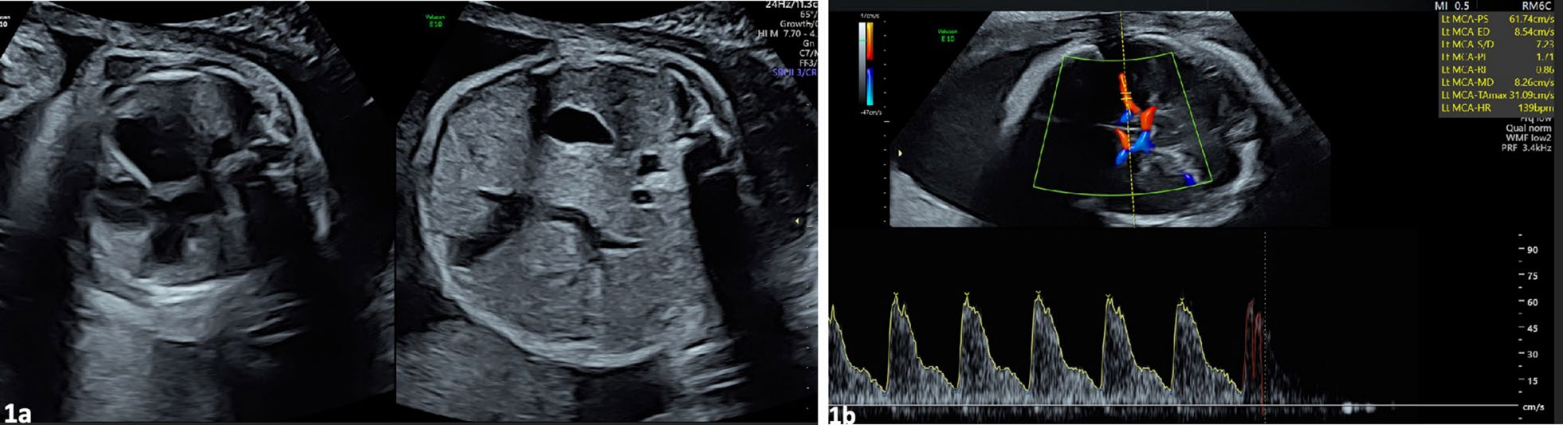
Ultrasound Diagnosis. **a** Transverse section of the fetal abdomen demonstrating cardiomegaly. **b** Transverse section of the brain at the level of the circle of Willis showing high MCA PSV

A 36-year old woman was referred to a tertiary unit at 29^+5^ weeks of gestation due to fetal cardiomegaly and mild ascites (Fig. 1a, b), which was detected on a scan undertaken for suspected small for gestational age. The woman was nulliparous with a low-risk first trimester combined screening test and unremarkable anomaly scan. She was rhesus B negative and underwent non-invasive prenatal testing that determined the rhesus genotype of the fetus to be rhesus B negative. Otherwise, no red blood cell antibodies were reported. Of note, the maternal grandmother was known to have sideroblastic anaemia that was diagnosed at 17 years of age. The mother of the unborn fetus and her brother had undergone testing as children but were told that no further follow-up was needed. Unfortunately, additional information regarding and genetic testing for this family history was not available. The woman had mild macrocytic anaemia with haemoglobin of 10.4 g/dl and a mean corpuscular volume of 104 fl. The woman had not received any preconceptional or genetic counselling.

**Fig. 2:**
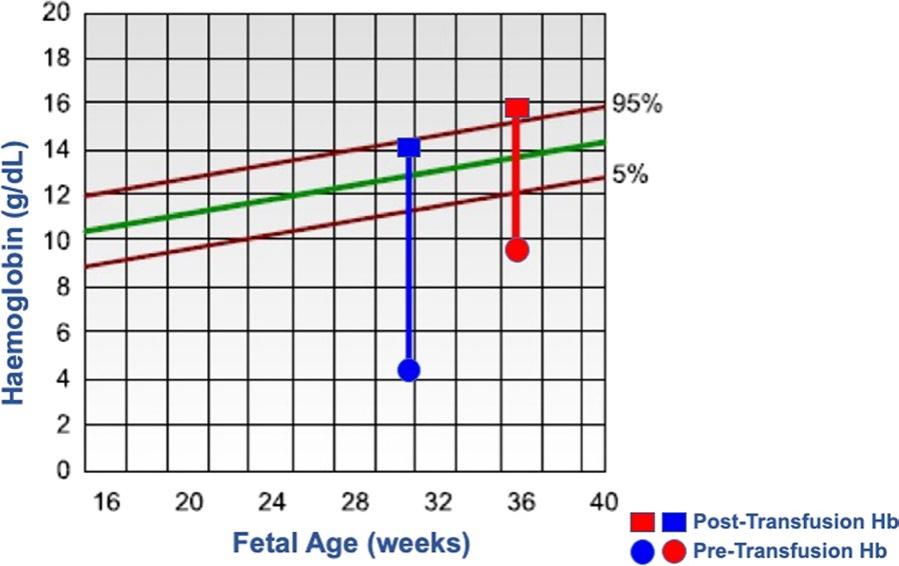
Haemoglobin levels before and after transfusion expressed in g/dL

Upon arrival, the first scan in our department demonstrated a middle cerebral arterial (MCA) peak systolic velocity (PSV) value above 1.5 Multiples of the Median (MoM). Fetal biometry was normal, as was the amniotic fluid index and umbilical artery doppler. Dexamethasone for fetal lung maturation was administered, and an uncomplicated in utero fetal blood transfusion (IUT) was performed at 30^+3^ weeks of gestation. The pre-transfusion fetal haemoglobin was 4.4 g/dl, which was increased to 14.1 g/dl following 120 mL of blood transfusion through the intrahepatic portion of the umbilical vein (Fig. 2). Investigations to determine the underlying cause of fetal anaemia included microarray comparative genomic hybridization, serology to exclude congenital infection, a peripheral blood film and fetal bilirubin to detect haemolysis. These preliminary investigations were normal. A fetal MRI brain was performed, which showed no abnormalities. In the subsequent weeks, the MCA-PSV improved, as did the cardiomegaly.

At 35^+3^ weeks of gestation, the MCA-PSV increased to above 1.5 MoM. The options of delivery and ex-utero transfusion versus IUT were discussed with the multidisciplinary team and with parents. As it was considered that an IUT would allow pregnancy to proceed to term, it was decided to proceed with IUT. A second IUT took place with a pre-and post-transfusion fetal haemoglobin of 9.7 and 15 g/dl, respectively. The MCA-PSV remained stable until 37 weeks and three days when a caesarean section for breech presentation was planned. Fetal biometry was consistent with previous measurements, and the amniotic fluid index and umbilical artery doppler were normal. The woman was counselled regarding the need for delivery and likely need for exchange transfusion in the neonatal period. Delivery of a female infant via caesarean section due to breech presentation was performed at 37 + 4 weeks. The birth weight was 3150 g, and apgar scores were 8 and 10 at 1 and 5 minutes, respectively.

Subsequently, the infant again became anaemic, requiring regular 3–4 monthly blood transfusions but she is making good developmental progress. Her anaemia was unresponsive to pyridoxine. Iron levels should be closely monitored to detect the need for chelation therapy in future care plans, since iron toxicity is a major cause of morbidity and mortality in XLSA [17].

**Fig. 3:**
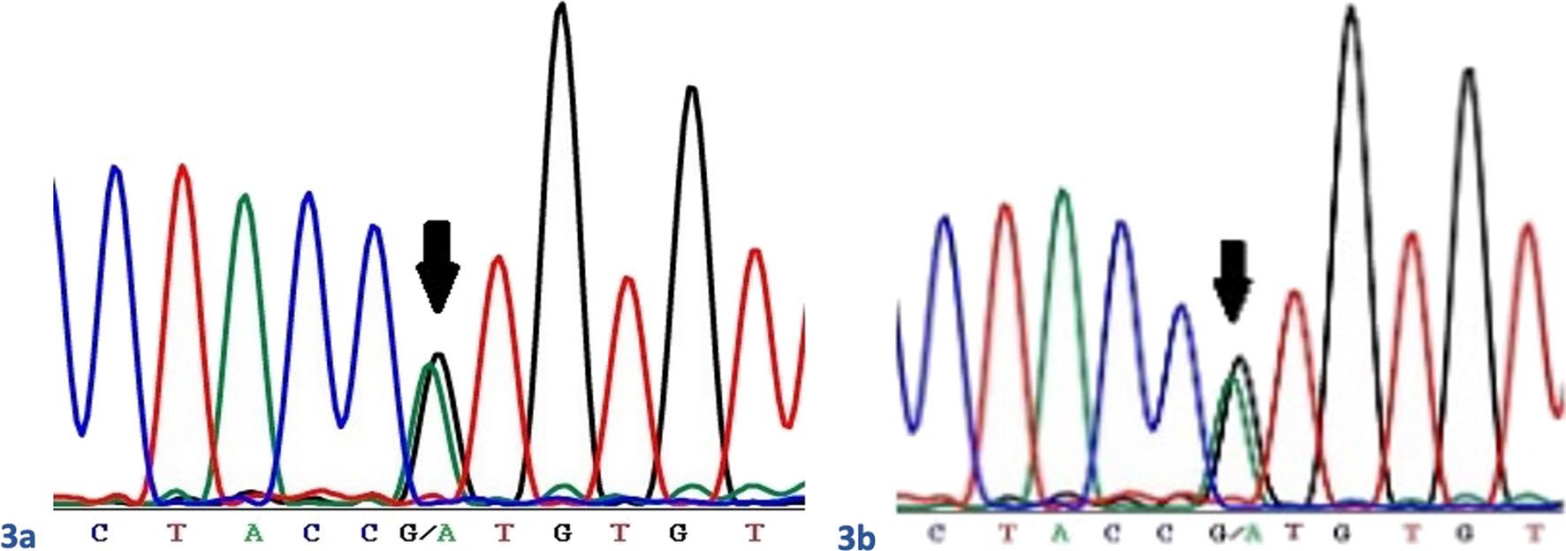
Sanger sequencing traces showing the heterozygous *ALAS2*c.488G > A; p.(Arg163His) variant. **a** In the mother and **b** in the fetus

Fetal and maternal blood was collected for DNA extraction, at the time of the first IUT. DNA was sequenced by next-generation sequencing for 11 genes associated with sideroblastic anaemia. Analysis was performed using Agilent SureSelect XT custom enrichment technology and Illumina DNA sequencing. Significant maternal cell contamination of the fetal blood sample was excluded using the ABI AMPFLSTR Identifiler PCR Amplification Kit. A heterozygous variant in the *ALAS2* (NM_000032.4) gene was identified in both the mother and the fetus. The c.488G > A; p.(Arg163His) variant identified affects a conserved amino acid and is absent from the gnomAD controls database [18]. The variant was therefore classified as pathogenic according to the American College of Medical Genetics (ACMG) variant interpretation guidelines [19]. The findings for both the mother and the fetus were confirmed by Sanger sequencing. These results were available two days following delivery and verified a heterozygous *ALAS2* c.488G > A; p.(Arg163His) and SLC4A1 c.876 + 5G > A mutations in both the baby (Fig. 3a) and the mother (Fig. 3b). X-inactivation studies [14, 20] were undertaken. There was no significantly skewed X-inactivation in the sample provided from the baby. The level of X-inactivation in the mother could not be determined, as she was uninformative for the *AR* locus [21].

## Discussion and conclusions

To our knowledge, this is the first case of anaemia reported in a female fetus heterozygous for an *ALAS2* mutation causing CSA. There is only one report in the literature of anaemia in a female fetus subsequently diagnosed with CSA, but, unlike our case, genetic analysis to determine the mutation was not performed on the infant or her family. Similar to our case, a woman presented at 28 weeks with features of fetal cardiomegaly and an enlarged liver and was managed with IUTs [22]. However, the diagnosis of sideroblastic anaemia in the female infant was made six weeks postnatally by bone marrow examination showing more than 50% of the erythroid cells with ringed sideroblasts. The mother had previously delivered a male fetus with severe anaemia and cardiac insufficiency, which resulted in neonatal demise 2 h after birth [22].

Although XLSA has predominantly been observed in hemizygous males, cases of several heterozygous female carriers of the *ALAS2* mutation have also been reported, often in mid to late adulthood [6, 7, 9]. X chromosome inactivation is the process by which the phenotype of heterozygous female carriers of the *ALAS2* mutation will be determined [14, 20]. The process of X chromosome inactivation in each cell is thought to be random such that most females will end up with a 50:50 mosaic expression of maternal and paternal X chromosomes. Under these circumstances, heterozygous female carriers of the *ALAS2* mutation will be asymptomatic. Some females will have mild abnormalities in their red cell indices because of variation from the 50% mean contribution of each X chromosome [23].

Families with more severe disease phenotype, with multiple females demonstrating extremely skewed x inactivation patterns, are rare. [6, 7, 9]. One hypothesis for this skewed X chromosome inactivation is that erythroid cells expressing the *ALAS2* mutation have a slight proliferative advantage over cells expressing the wild type allele. Ultimately, erythroid cells with an inactivated normal X chromosome gradually become more predominant over time, resulting in the progression of anaemia with age [24]. Observations made by Aivado et al. support this hypothesis where a female known to be heterozygous for *ALAS2* R436W mutation became progressively anaemic in her sixth decade of life. The fall in her haemoglobin coincided with an increase in the percentage of bone marrow erythroid cells with an inactivated normal X chromosome [7].

It remains unknown as to what determines the rate of progression of X chromosome inactivation and, thus, the clinical course of disease in females. For example, in our case, the maternal grandmother, despite having had the mutation, only became symptomatic at the age of 17. This is in contrast to her granddaughter, who was severely affected in utero and her daughter, also heterozygous and approaching the fourth decade of life, who has remained asymptomatic. Several mechanisms controlling the progression of X chromosome inactivation have been proposed. These include age-related depletion of hematopoietic stem cells [24], structural abnormalities in the X chromosome [25] or a mutation in the promoter region of the X chromosome inactivation specific transcript (XIST) gene whose non-coding RNA product typically maintains X chromosome inactivation [25].

There are three other case reports describing families in which multiple heterozygous females have been affected by XLSA of varying severity (Table 1). One case report was of a family in which a 42-year old woman presented with macrocytic anaemia and splenomegaly. The woman, her mother and possibly her sister were all found to be heterozygous for c.679C > T mutation in exon 6 of the *ALAS2* gene [6]. The second case report describes a family with three daughters. The mother and the eldest daughter were first noted to be anaemic at the age of 16 and 41 years, respectively, and later became transfusion dependent. The middle daughter was unaffected, and the youngest daughter had mild anaemia, not requiring transfusions. The mother and the two affected daughters were heterozygous for c.1358C > T mutation in exon 9 of the *ALAS2* gene. Interestingly, in this latter study, DNA sequencing excluded a mutation of the XIST gene promoter region in the mother and affected daughters as a possible cause for the skewed X chromosome inactivation [7]. The final case report is of a 32-year old woman with macrocytic anaemia and iron overload who, along with her sister and mother, both also with macrocytic anaemia, were found to be heterozygous for Y365C mutation in the *ALAS2* gene [9]. It is notable that in all families, such as that reported here, where there are multiple female members with XLSA, affected male members have not been encountered. This suggests that, first, the type of *ALAS2* mutation may be an important determinant of disease expression in women. Second, a mutation leading to disease in females is lethal in an affected male conceptus.

**Table 1:** Comparison of XLSA where multiple female members in the same family were affected (1–3) to the case in this report (4)

**Case**	1	2	3	4
Age at diagnosis	61	42	32	*In-Utero*
Other family Members affected	Mother	Mother	Mother	Mother and grandmother
ALAS2 mutation	R163H	R227C	Y365C	R163H
Pyridoxine responsive	No	No	Not known	No
Skewed X-inactivation	No	Not known	Not known	No
References	[15]	[9]	[12]	

The p.Arg163His *ALAS2* mutation has previously been reported in a 61-year old woman presenting with macrocytic anaemia and iron overload [12]. Her mother also had a history of anaemia but was not tested for the mutation. In this study, in vitro analysis with bacterially expressed recombinant *ALAS2* protein confirmed that the p.Arg163His mutation severely diminished its enzymatic activity [12]. The *SLC4A1* c.876 + 5G > A mutation has not previously been reported to cause anaemia in the literature and occurs at a frequency of 0.0009% in the European population (gnomAD database). The impact of this latter mutation on the phenotype of both the mother and fetus remains unknown, as it is commonly associated with autosomal dominant red cell membranopathies [26]. In the absence of a significantly skewed X chromosome inactivation in our case, one hypothesis would be a possible interaction between the dysfunctional *SLC4A1* protein and the *ALAS2* protein.

For counselling patients and families, the ratio of X inactivation could help predict the potential course of the disease. In a study of nine women with XLSA heterozygous for *ALAS2* mutation, their X inactivation ratios ranged from 99:1 to 77:23. In comparison, the X inactivation ratios in 10 unrelated asymptomatic carriers ranged from 50:50 to 68:32 [27]. This is consistent with observations from Aivado et al. and Sankaran et al., who both concluded that the disease severity was proportionate to the degree of X chromosome inactivation [7, 9]. However, our case highlights the uncertainty of the recurrence risk in a subsequent pregnancy. If the fetus is male, then there is a 50% risk of inheriting and being affected by this mutation that may potentially be lethal. If the fetus is female, then there is a 50% risk of inheriting this mutation and at least being a carrier. However, it is impossible to predict the pattern of disease severity that may manifest either in utero or later in life. Based on the available literature, the mother may be reassured that the majority of carrier females would, if at all, manifest symptoms later in life. Still, close ultrasound observation in utero should be advised.

In addition, It is important to recognize and alert clinicians that female XLSA patients are sometimes mis-diagnosed as having a myelodysplastic syndrome since many present with macrocytic or normocytic RBCs and are usually refractory to pyridoxine supplementation. Nonetheless, a significant number of female XLSA patients are, like male XLSA patients, microcytic and usually pyridoxine-responsive to varying degrees [17].

This is one of four cases [6, 9, 12] reporting multiple female members presenting with discordant clinical features of XLSA from being entirely asymptomatic to anaemic in utero. There are no other reports in the literature of anaemia in a female fetus heterozygous for *ALAS2* mutation.

## Data Availability

The details of the variant analyzed during the current study are available in the ClinVar repository, under the Accession Number SCV001573241.1. The raw datasets generated during the current study are not publicly available because it is possible that individual privacy could be compromised. It is possible to apply for permission to obtain access to the raw sequencing data and the details of the postmortem examination through the corresponding author.

## Abbreviations

ACMG: American college of medical genetics
CSA: Congenital sideroblastic anaemia
DNA: Deoxyribonucleic Acid
IUT: In utero fetal blood transfusion
MCA: Middle cerebral artery
MCV: Mean corpuscular volume
MoM: Multiples of the Median
PSV: Peak systolic velocity
XIST: X chromosome Inactivation Specific Transcript
XLSA: X-linked sideroblastic anaemia
